# Research Capacity and Culture Development in a Small Rural Health Service

**DOI:** 10.1111/ajr.70165

**Published:** 2026-03-09

**Authors:** Dai Pu, Stephen Gill, Michael Field, Alison Buccheri, Olivia A. King, Catherine E. Huggins, Terry Haines, Joanne E. Porter, Vincent L. Versace, Laura Alston

**Affiliations:** ^1^ School of Primary and Allied Health Care, Faculty of Medicine, Nursing and Health Sciences, Monash University Victoria Australia; ^2^ Barwon Centre for Orthopaedic Research and Education (BCORE), Deakin University, St John of God Hospital Geelong and Barwon Health Geelong Victoria Australia; ^3^ Research Unit, Colac Area Health Australia; ^4^ Deakin Rural Health School of Medicine, Deakin University Melbourne Australia; ^5^ Western Alliance Academic Health Science Centre Warrnambool Australia; ^6^ Barwon Health Geelong Australia; ^7^ Institute of Health Transformation, School of Health and Social Development Deakin University Geelong Australia; ^8^ Collaborative Evaluation and Research Centre (CERC) Federation University Australia Victoria Australia; ^9^ Centre for Australian Research Into Access Deakin University Warrnambool Australia

**Keywords:** capacity building, health workforce, professional competence, rural health, work force

## Abstract

**Objective:**

This study aimed to measure changes in staff perceptions of research capacity and culture in a small rural health service in Australia over time.

**Methods:**

Staff completed the Research Capacity and Culture Tool, a valid and reliable survey that measures individuals' perceptions of their own research capacity and the research capacity and culture of their team and organisation. Data from 2015 was compared to 2023, following significant changes at the health service that focused on integrating research into the organisational structure.

**Design:**

This was a repeated cross‐sectional study in which data were collected from different individuals.

**Setting:**

Data were collected from a rural health service in Victoria (Modified Monash Model 4–5).

**Participants:**

All staff working in the health service were invited to complete the survey.

**Main Outcome Measures:**

Research Capacity and Culture Tool.

**Results:**

Results demonstrated improvements in eleven out of eighteen measures of research capacity and culture at the organisation level, six out of nineteen measures at the team level, but none at the individual level. Median improvements were modest, typically two points on the 10‐point scale.

**Conclusions:**

Integrating research into the health service organisation structure may be beneficial for its research capacity and culture.

## Abstract (highlights)

**What This Paper Adds:**

- Strategies such as having an embedded research unit, local research policies, and strong external research partnerships might improve research capacity and culture in rural health service settings.
- Research units embedded in health services may be beneficial for improving organisational and team level research culture and capacity, but individual research skills may require additional strategies to build over time.

**What Is Already Known on This Subject:**

- High levels of research capacity and a strong research culture are associated with better patient outcomes and staff satisfaction in health services.
- Improving research capacity and culture in rural health services is difficult due to the unique challenges, such as workforce fragmentation and shortages, and limited research infrastructure.
- It is important to monitor and measure research capacity and culture periodically to capture evolving health service research culture and the effectiveness of any programs.

## 1 Introduction

Research capacity and culture can influence the clinical and public health practices of an organisation [1, 2, 3]. Good clinical practice relies on the accurate understanding, interpretation, and translation of research evidence. At the same time, the knowledge and skills needed to design and conduct scientifically sound research are important for clinicians and their organisations to contribute to evidence‐based practice. Understanding the research skill levels and the supports required will identify the training needs in an organisation and inform targeted strategies to address these needs.

Research capacity building has been defined as a process of developing sustainable abilities and skills enabling individuals and organisations to perform high quality research [4]; and research culture can be considered the “research environment” [4] that supports the research and can be indicative of the value placed on research by the organisation. Research capacity is dynamic and can be observed at multiple levels of a health care organisation, including individuals, teams and departments [5].

Programs designed to improve health service research capacity and culture can struggle with sustainability due to lack of funding and inconsistent evaluation [6]. Key to ensuring ongoing progress and improvement is to systematically track and evaluate these investments and their outcomes [6, 7].

Research capacity in rural and regional areas is consistently lower compared to metropolitan areas where large research universities and health services are concentrated [3, 8, 9, 10]. Research education is also less accessible in these areas [9]. Without intervention, research culture and capacity can remain stagnant over time [11]. Increasingly, rural health services are building research into their organisations to improve research capacity and culture and attract and retain critical health workforce. Colac Area Health (CAH) provides health care services to communities in areas classified as MM4 and MM5 by the Modified Monash Model. Since 2020, the organisation has established a research unit, which integrated research into the organisational structure, strengthened by external partnerships with the local University Department of Rural Health (Deakin Rural Health) and the Western Alliance Academic Health Science Centre, and has been described in detail elsewhere [12, 13, 14, 15]. In short, the research unit was included in the organisational structure and the strategic plan, with direct reporting to the CEO via the research unit director. Its aim was to support staff to develop their research skills and ideas, as well as house research staff leading externally funded projects at CAH. The research unit started with two staff members in 2020 and has since included up to 8 staff members depending on research funding.

This study aimed to measure changes in staff perceptions of the research capacity and culture at CAH over time.

## 2 Materials & Methods

Research capacity and culture was compared across two time points. Historical data was obtained from a study that used the Research Capacity and Culture (RCC) Tool [4] to survey eight health services in Victoria in 2015 [3]; RCC data was collected at one of these health services again in 2023 [13]. Both studies were approved by Barwon Health Human Research Ethics Committee (2015 reference: 14/136; 2023 reference: 23/29), with additional approvals for data sharing and comparisons for this study.

### 2.1 Participants and Setting

Data were collected from CAH, a rural health service that serves a region classified as small‐medium rural towns on the Modified Monash Model [16] (MM4‐5). CAH services a population of approximately 32 000 via one urgent care centre, 28 inpatient beds that encompass medical, surgical and maternity care, specialist clinics, aged care, and community health services [17]. At both time points of data collection, all staff in the health service were invited to participate. There were 361 non‐casual staff members employed at CAH in 2015, and 598 staff in 2023.

### 2.2 Survey Tool

The RCC tool [4] was used to measure research capacity and culture. The RCC tool is based on the research capacity building framework proposed by Cooke in 2005 [18], and asks health service staff 55 questions: 20 questions each for how they perceived their team and organisation's research capacity and culture, and 15 questions for how they perceived their own research skills. Each question asks for a rating from 1 (least success) to 10 (most success). The 2015 survey excluded four questions: one question about individual research capacity (You integrate research findings into practice), one question about team‐level research culture (Your team provides access to literature searching and article retrieval), and two questions about organisational research culture (Your organisation provides access to literature searching and article retrieval and Your organisation requires ethics approval for research activities). A total of 51 questions were compared between the two survey rounds.

### 2.3 Data Collection

The survey was distributed online using REDCap at both timepoints. Recruitment was conducted via emails sent to individual staff members' organisation email. One email was sent to invite staff to complete the survey in 2015; up to two emails were sent in 2023. Respondents did not have to provide a rating for every RCC question if they chose not to.

### 2.4 Analysis

Ratings for RCC tool questions were described as medians and interquartile ranges. Comparisons across time were conducted using the non‐parametric Kruskal–Wallis test. Paired analysis was not conducted as individuals were not matched across time. Holm's–Bonferroni corrections were used to determine statistical significance for each level of the RCC Tool to account for the higher probability of Type 1 errors due to multiple comparisons.

## 3 Results

In the 2015 dataset, there were 50 responses (13.9%) from a total of 361 non‐casual staff members who were sent the email inviting survey completion; in the 2023 dataset, there were 74 responses (12.4%) from a total of 598 staff employed by CAH. Respondent characteristics are reported in Table 1.

**TABLE 1:** Respondent characteristics.

	2015 (*n* = 50)	2023 (*n* = 74)
**Gender** Male Female Unreported	3 (6.0%) 33 (66.0%) 14 (28.0%)	10 (13.5%) 56 (75.7%) 8 (10.8%)
**Field of Employment** Clinical Staff Management Administration Others/Unreported	31 (62.0%) 0 4 (8.0%) 15 (30.0%)	35 (47.3%) 7 (9.5%) 14 (19%) 18 (24.3%)

From 2015 to 2023, CAH staff reported perceived improvements in 11 out of 18 measures of research capacity and culture at the organisation level, 6 out of 19 measures at the team level, but none at the individual level (Table 2). Significant improvements were most commonly a median of two points. One item (My organisation provides experts for research advice) demonstrated a median improvement of 5 points. No deteriorations were observed for any measures.

**TABLE 2:** Changes in RCC across time.

	2015 (*N* = 50)	2023 (*N* = 74)	*p*
	**median (interquartile range) (n)**		
**My organisation**			
provides resources to support staff research training.	5 (3–7) (*n* = 50)	6 (4–7.5) (*n* = 45)	*p* = 0.158
provides funds, equipment or admin to support research activities.	4 (2–7) (*n* = 49)	5 (4–8) (*n* = 45)	*p* = 0.013
has a plan or policy for research development.*	5 (2.25–7) (*n* = 48)	7 (5–9) (*n* = 45)	*p < *0.001
provides access to literature search and article retrieval.	N/A	6 (4–8.5) (*n* = 45)	N/A
has senior managers that support research.	6 (3–8) (*n* = 48)	7 (5–9) (*n* = 45)	*p* = 0.013
ensures staff career pathways are available in research.*	4 (2–7) (*n* = 48)	5 (4.5–8) (*n* = 45)	*p* = 0.002
ensures organisation planning is guided by evidence.	6 (4–8) (*n* = 48)	7 (5–8) (*n* = 44)	*p* = 0.611
has consumers involved in research.	5.5 (3–7) (*n* = 48)	7 (5–8) (*n* = 43)	*p* = 0.041
accesses external funding for research.*	5 (2–7) (*n* = 47)	7 (5–9) (*n* = 43)	*p* < 0.001
promotes clinical practice based on evidence.	7 (5–8) (*n* = 47)	7.5 (6–9) (*n* = 44)	*p* = 0.149
encourages research activities relevant to practice.*	5 (3–7) (*n* = 46)	7 (5–9) (*n* = 43)	*p* < 0.001
provides software programs for analysing research data.	3 (2–6) (*n* = 47)	5 (4–7) (*n* = 43)	*p* = 0.033
has mechanisms to monitor research quality.*	4 (2–7) (*n* = 45)	6 (5–8) (*n* = 44)	*p* = 0.002
provides experts for research advice.*	2 (1.5–6) (*n* = 45)	7 (5–9) (*n* = 44)	*p* < 0.001
supports a multi‐disciplinary approach to research.*	5 (2–7) (*n* = 46)	6.5 (5–9) (*n* = 44)	*p* < 0.001
provides forums or bulletins to present research findings.*	5 (1–7) (*n* = 46)	7 (5.25–9) (*n* = 44)	*p* < 0.001
engages external partners (e.g., Universities) in research.*	5 (1.5–7) (*n* = 45)	8 (6–9) (*n* = 43)	*p* < 0.001
supports applications for research scholarships or degrees.*	5 (3–7) (*n* = 45)	6.5 (5–9) (*n* = 44)	*p = 0*.002
supports the peer‐reviewed publication of research.*	5 (2–7) (*n* = 45)	7 (5–9) (*n* = 44)	*p* < 0.001
requires ethics approval for research activities.	N/A	8.5 (7–10) (*n* = 44)	N/A
**My team**			
provides resources to support staff research training.**	4 (1.25–6.75) (*n* = 40)	6 (5–8) (*n* = 41)	*p* = 0.001
provides funds, equipment or administration to support research activities.**	3.5 (1–6) (*n* = 40)	5 (5–7.5) (*n* = 41)	*p < 0*.001
does team level planning for research development.	4 (2–6) (*n* = 40)	5 (5–7.5) (*n* = 41)	*p* = 0.007
ensures staff involvement in developing that plan.	4 (2–7) (*n* = 40)	5 (5–8) (*n* = 41)	*p* = 0.022
has team leaders that support research.	5 (3–7) (*n* = 39)	6 (5–8) (*n* = 41)	*p* = 0.013
provides opportunities to get involved in research.**	4 (1.25–7) (*n* = 40)	6 (5–8) (*n* = 41)	*p* = 0.003
does planning that is guided by evidence.	6 (4–8) (*n* = 40)	7 (5–8) (*n* = 41)	*p* = 0.124
has consumer involvement in research activities or planning.	5 (2–8) (*n* = 40)	5 (5–7.75) (*n* = 40)	*p* = 0.198
has applied for external funding for research.**	4 (1–6) (*n* = 40)	5.5 (4–8) (*n* = 40)	*p* = 0.003
provides access to literature searching and article retrieval.	N/A	5 (3.5–8) (*n* = 41)	N/A
conducts research activities relevant to practice.	4 (1.25–7) (*n* = 40)	6 (4.5–8) (*n* = 41)	*p* = 0.020
supports applications to research scholarships or degrees.	5 (3–7) (*n* = 40)	5 (5–8) (*n* = 41)	*p* = 0.171
has mechanisms to monitor research quality.	3.5 (1–7) (*n* = 40)	5 (4–8) (*n* = 41)	*p* = 0.022
provides experts accessible for research advice.**	3 (1–6.75) (*n* = 40)	5 (5–8) (*n* = 41)	*p < 0*.001
disseminates research results at research forums or seminars.**	4 (1–7) (*n* = 40)	6 (5–8) (*n* = 41)	*p < 0*.001
supports a multi‐disciplinary approach to research.	5 (2–7) (*n* = 40)	5 (5–8) (*n* = 41)	*p* = 0.013
has incentives and support for mentoring activities.	4 (2–7) (*n* = 40)	5 (5–7) (*n* = 38)	*p* = 0.026
has external partners (e.g., universities engaged in research).	4.5 (2–7) (*n* = 40)	5 (5–8) (*n* = 41)	*p* = 0.017
supports peer‐reviewed publication of research.	5 (1.25–7) (*n* = 40)	5 (5–8) (*n* = 40)	*p* = 0.007
provides software to support research activities.	3 (1–7) (*n* = 39)	5 (3–6) (*n* = 40)	*p* = 0.040
**I**			
find relevant literature.	8 (6–8) (*n* = 37)	8 (6–9) (*n* = 39)	*p* = 0.895
critically review the literature.	7 (4–8) (*n* = 37)	7 (5–8) (*n* = 39)	*p* = 0.252
use a computer referencing system (e.g., Endnote).	6 (2–8) (*n* = 37)	5 (3–8) (*n* = 38)	*p* = 0.593
write a research protocol.	5 (2–6.5) (*n* = 37)	5 (3–8) (*n* = 35)	*p* = 0.119
secure research funding.	3 (2–5.75) (*n* = 36)	5 (2–8) (*n* = 35)	*p* = 0.022
submit an ethics application.	3 (1.25–5.75) (*n* = 36)	5 (2–8) (*n* = 36)	*p* = 0.028
design questionnaires.	6 (2.5–7.5) (*n* = 37)	7 (5–9) (*n* = 38)	*p* = 0.024
collect data, e.g., surveys, interviews.	7 (3.5–8) (*n* = 37)	7 (5–9) (*n* = 37)	*p* = 0.195
use computer data management systems.	6 (2–8) (*n* = 37)	6 (5–9) (*n* = 36)	*p* = 0.149
analyse qualitative research data.	5 (2.5–7) (*n* = 37)	7 (5–9) (*n* = 35)	*p* = 0.017
analyse quantitative research data.	5 (2.25–7) (*n* = 36)	6.5 (3.5–8) (*n* = 36)	*p* = 0.039
write a research report.	6 (2–7) (*n* = 36)	6 (3–8) (*n* = 36)	*p* = 0.094
write for publication in peer‐reviewed journal.	2.5 (1–6) (*n* = 36)	5 (2.25–7.75) (*n* = 36)	*p* = 0.025
integrate research findings into practice.	N/A	7 (5–8) (*n* = 36)	N/A
provide advice to less experienced researchers.	3 (1–6) (*n* = 36)	5 (3.25–8) (*n* = 36)	*p* = 0.011

* Statistical significance at the organisation level achieved at *p* = 0.006.

** Statistical significance at the team level achieved at *p* = 0.004.

## 4 Discussion

This study found improvements in research capacity and culture over time in a rural health service in Victoria, after significant efforts were made to integrate research into the organisation. These improvements were reported primarily for items at the team and organisation levels, with non‐significant changes at the individual level. While team and organisational improvements were statistically significant, these changes were typically modest in size and between 1 to 3 points. The largest improvement was reported for CAH's provision of experts for research advice, which increased from a median rating of 2 to 7. This improvement could be the consequence of introducing the research unit which increased the availability of research experts to provide advice to the organisation.

The introduction of an embedded research unit in 2020 dedicated to supporting research activities in the rural setting [12, 15, 19] may have contributed to improvements to research capacity and culture. The 2023 dataset was analysed and reported in a previous study [13], which found that staff who had received support from the research unit reported higher ratings for organisational research culture *and* individual research capacity compared to those who had not [13]. This result was similar to a study that reported the introduction of a dedicated research support office was associated with increased individual research capacity in a metropolitan health service [20]. In contrast, the current comparison across time only found change at the organisational and team levels, but not the individual level. This might be due to a few reasons. First, individual RCC levels have tended to be higher than team and organisation levels in previous studies [10], indicating a possible ceiling effect. Second, the high turnover rate in more remote health services [8] could mean that the same staff who might have reported improvements in research skills were not captured after leaving the health service. Finally, a research unit integrated into the organisational structure with supporting policies and locally employed staff may be most effective in improving research culture at higher levels, but benefits may not always filter to team/departmental and individual levels in short time periods [15].

Future research should monitor the reach and effects of having integrated research units in rural health services. This could continue to utilise the RCC tool survey used in the current study, as well as qualitative interviews to gather more detailed information from staff. Traditional research metrics such as the number of peer‐reviewed publications and grants received could also be used to evaluate the effects of initiatives to improve research culture and capacity.

## 5 Limitations

Data used in the current study was limited to a single rural health service, limiting the generalisability of the findings. The RCC tool measures staff‐perceived research culture and capacity, which is subjective and may not be reflective of the actual research support infrastructure and processes in place. While associations may be drawn between the embedded research unit in CAH and improvements in staff‐perceived research capacity and culture, no direct causal relationship can be concluded. The repeated cross‐sectional design of this study means that the same participants were not followed over time, so the observed changes could be due to the characteristics of each sample rather than changes over time. While the survey was sent to all health service staff and response rates were similar across time (13.9% in 2015 and 12.4% in 2023), it is possible that only those with an interest and active engagement in research activities responded to the survey, thereby potentially limiting the representativeness of the findings.

## 6 Conclusion

Embedded research units that support research conduct in rural health services may be a promising approach to supporting the growth of research culture and capacity for the rural health workforce.

## Author Contributions

**Alison Buccheri:** data curation, methodology, writing – review and editing. **Laura Alston:** data curation, methodology, writing – review and editing. **Vincent L. Versace:** data curation, writing – review and editing. **Terry Haines:** methodology, writing – review and editing. **Dai Pu:** conceptualization, data curation, formal analysis, investigation, methodology, project administration, writing – original draft, writing – review and editing.

## Funding

This work was supported by The Medical Research Future Fund, RARUR000072.

## Disclosure

The data used in this study were previously published in two different papers that reported single timepoint analyses.

## Conflicts of Interest

Authors Alston, Field and Buccheri have roles within the health services from which this study's data was collected. Data analysis was conducted by Dai Pu, who has no current or prior relationship with these health services to address this conflicts of interest.

## Data Availability

Data sharing not applicable to this article as no datasets were generated or analysed during the current study.
